# Biochar and Natural Antioxidants as Components of Eco-Friendly Elastomer Composites

**DOI:** 10.3390/polym17172351

**Published:** 2025-08-29

**Authors:** Justyna Miedzianowska-Masłowska, Kalina Joanna Kaczmarek, Marcin Masłowski

**Affiliations:** Institute of Polymer and Dye Technology, Lodz University of Technology, Stefanowskiego 16, 90-537 Lodz, Poland; 243210@edu.p.lodz.pl (K.J.K.); marcin.maslowski@p.lodz.pl (M.M.)

**Keywords:** natural rubber, biochar, natural antioxidants, biocomposites, functional properties

## Abstract

Modern trends in advanced material design increasingly emphasize sustainability and the use of naturally derived resources. One promising approach involves replacing synthetic additives with natural compounds that exhibit stabilizing properties. The aim of this study was to evaluate the effects of selected natural auxiliary substances—thymol (2-isopropyl-5-methylphenol), quercetin (3,3,4,5,7-pentahydroxyflavone) and caffeic acid (3-(3,4-dihydroxyphenyl)prop-2-enoic acid)—on the properties of elastomeric composites based on natural rubber. Biochar was used as the filler in the composites, serving as an eco-friendly alternative to conventional carbon black. The evaluation included measurements of crosslink density, hardness, mechanical properties and microstructural analysis of the resulting materials. The samples were also subjected to accelerated aging under thermo-oxidative conditions and UV radiation to assess their resistance to degradation. For comparison, the commonly used synthetic antioxidant BHT (2,6-di-tert-butyl-4-methylphenol) was also analyzed. The results enabled the assessment of the potential of natural additives as environmentally friendly stabilizers in elastomeric systems, with respect to their effectiveness and impact on material durability.

## 1. Introduction

Elastomers are gaining increasing importance in modern polymer-processing industries due to their unique combination of flexibility, abrasion resistance and insulating properties [1]. Their molecular architecture, characterized by long, loosely arranged polymer chains interconnected by cross-links, enables reversible deformation under stress [2]. These characteristics make them indispensable in a wide range of applications, including automotive, construction and biomedical sectors [1,3]. However, the continuous advancement of technology and increasingly demanding service conditions necessitate the development of elastomeric materials with improved durability, enhanced thermal and chemical stability and extended operational lifespans [4,5,6]. Increasing environmental awareness and the need to reduce industrial impact on the ecosystem are driving the development of sustainable elastomeric materials [7,8,9]. There is a growing shift toward replacing conventional synthetic additives with renewable, biodegradable or recycled resources [10]. The goal is not only to lower the environmental footprint but also to enhance performance by incorporating eco-friendly fillers and additives that improve the durability and efficiency of elastomers throughout their entire life cycle [11].

One promising direction in sustainable material development is the use of biochar as an environmentally friendly alternative filler in rubber composites [12,13,14,15,16,17,18,19]. Biochar, produced through the pyrolysis of biomass (e.g., plant waste), offers an attractive substitute for conventional carbon black, which is derived from non-renewable petroleum-based sources. In addition to reducing environmental impact, biochar can modify the mechanical and thermal properties of elastomers, influencing parameters such as hardness, abrasion resistance and thermal conductivity [20,21]. Moreover, it has the potential to enhance tensile strength, improve elastic recovery and modify dynamic mechanical behavior, contributing to overall performance optimization [22,23,24,25]. As such, it aligns with the principles of green chemistry and represents a significant step toward more sustainable material technologies.

Biochar-filled elastomer composites represent a promising class of materials with considerable potential in the context of sustainable polymer processing. As products of biomass pyrolysis, biochars are not composed solely of carbonaceous material but also contain a wide range of inorganic constituents [26]. These include substantial amounts of minerals such as Ca, K, Mg, Si, Na, Fe, Mn, Cu and Zn, primarily present as oxides, carbonates and phosphates [27,28]. The presence and concentration of these elements are largely influenced by the type of biomass used and the conditions under which pyrolysis is carried out [29]. While such mineral inclusions can positively affect certain performance aspects of the composites, they also introduce challenges related to stability. Specifically, some of these components may function as catalysts or initiators of degradation reactions under thermal and oxidative conditions, thereby accelerating material aging and reducing the overall service life of the composites [30,31,32]. Consequently, it is essential to develop and implement effective stabilization strategies to enhance environmental resistance and ensure long-term durability in technical applications.

In the elastomer industry, synthetic stabilizers are commonly used as antioxidants to protect materials from oxidative and thermal degradation [33]. While the effectiveness of these additives is well documented, increasing attention is being paid to their potential adverse effects on human health and the environment [34,35]. Such compounds can migrate from finished products, accumulate in living organisms and contribute to the contamination of aquatic and soil ecosystems [36,37]. Consequently, there is growing interest in naturally derived substances—such as plant extracts and polyphenols—which can function as stabilizers in a more environmentally friendly manner, while maintaining their effectiveness within elastomeric matrices [38,39,40].

The aim of this study is to evaluate the potential application of selected naturally derived compounds: thymol, quercetin and caffeic acid as modifiers of elastomer composites, with particular emphasis on their stabilizing capabilities. These compounds, belonging to the group of natural antioxidants, exhibit the ability to chelate heavy metal ions [41,42,43], thereby mitigating catalytic oxidative degradation processes within the polymer matrix. The proposed research hypothesis posits that the use of these natural additives may provide stabilization efficiency comparable to or exceeding that of conventional synthetic antioxidants, such as butylated hydroxytoluene (BHT), while simultaneously enhancing the environmental friendliness and safety of the final products.

The results obtained may represent an important step towards the development of more sustainable and environmentally friendly elastomeric composites, in which traditional synthetic additives are replaced by substances of natural origin. Demonstrating the effectiveness of compounds such as thymol, quercetin or caffeic acid as stabilizers not only supports the advancement of green chemistry but also expands the current understanding of the mechanisms of action of natural antioxidants in polymer systems.

## 2. Materials and Methods

### 2.1. Preparation of the Bio-Sourced Filler

#### Bio-Sourced Filler

Biochar for natural rubber composites was sourced from wheat straw. It was subjected to a grinding process in a Robot-Coupe Blixer 4 blender (Robot-Coupe S.N.C., Vincennes, France) for a duration of 10 min, after which the straw was ground into a meal in a Mixer/Mill 80,000 ball mill, a process which took 20 min.

The material thus prepared was then subjected to a pyrolysis process in a ceramic vessel. This stage was maintained for a period of 2 h under a nitrogen gas flow of 200 mL/min and heated to 600 °C. The amount of biochar used in the natural rubber composites was fixed at 20 phr based on our previous research and experimental experience [12]. This particular loading was found to provide a good balance between processing properties and mechanical performance of the vulcanizates.

### 2.2. Composition of the Mixtures

Natural rubber (NR)—RSS 1 (Ribbed Smoked Sheet 1), primarily composed of *cis*-1,4-polyisoprene with a density of 0.93–0.98 g/cm^3^, was provided by Torimex Group Sp. z o.o. (Konstantynów Łódzki, Poland). The material originated from Thailand. This elastomer was vulcanized with sulfur, microsized zinc oxide and stearic acid as the standard activators and 2-mercaptobenzothiazole (MBT) as an accelerator. The compositions of the mixtures used for further tests are presented in Table 1.

The compound preparation process was divided into two stages. In the first stage, a premix containing natural rubber (in the form of small pieces), filler, and anti-aging agents (in powder form) was prepared. The following substances were used as anti-aging agents: thymol (TYM), quercetin (QR), caffeic acid (CA), and butylated hydroxytoluene (BHT). The formulations of all compounds are expressed in phr (parts per hundred parts of rubber), which is a standard notation in rubber technology. In practice, this means that each batch was prepared with 100 g of natural rubber, and the respective amounts of other ingredients were calculated accordingly, as shown in Table 1.

A Brabender micromixer was used for compounding. In the first step, the raw rubber was preliminarily plasticized for approximately 5 min through mastication under shear and temperature, which facilitated subsequent dispersion of the additives. Next, the powdered ingredients were gradually incorporated. All additives were dried prior to mixing to prevent the influence of moisture on dispersion quality. The total mixing time for one batch was approximately 15 min. The processing conditions (50 °C, 40 rpm) were optimized to allow sufficient softening of the rubber matrix while ensuring homogeneous distribution of the additives under laboratory-scale conditions. The adequacy of these parameters was confirmed by the uniform appearance of the mixtures and the reproducibility of results across all prepared batches.

In the second stage of the process, the crosslinking unit was introduced on a laboratory two-roll mill, which simultaneously homogenized the compounds further and formed them into flat sheets suitable for subsequent testing.

Following the completion of rheometric tests and differential scanning calorimetry, the prepared blends were subjected to the crosslinking process. Fourteen grams of each blend was weighed out, placed between layers of Teflon film, and then pressed at 160 °C for 11 min. In this way, vulcanized samples in the form of thin plates with a thickness of 1.0 ± 0.2 mm were obtained, suitable for subsequent testing.

### 2.3. Rheometric Properties of the Mixtures

The experimental procedure was carried out using a MonTech non-rotating rotational rheometer (MonTech GmbH, Columbia City, IN, USA), model D-RPA 3000. Samples were prepared by cutting small pieces from the non-crosslinked material, which were then inserted between transparent films and placed in the rheometer. The measurement was then conducted for a duration of 20 min at the designated temperature of 160 °C.

The purpose of this test was to determine the torque increase during the vulcanization of the mixture. The test facilitated the determination of the torque increase during mixture vulcanization by using the following Equation (1):

(1) $$∆M=M_{max}-M_{min}$$

∆M—torque increase during mixture vulcanization [dNm]; $M_{max}$—maximum torque during mixture vulcanization. $M_{min}$—minimum torque during mixture vulcanization.

### 2.4. Determination of Vulcanization Kinetics

DSC measurements were performed using a Mettler Toledo DSC1 calorimeter (Mettler-Toledo International Inc., Greifensee, Switzerland). Approximately 8 mg of the non-cross-linked rubber mixture was placed in specialized calorimetric pans, accompanied by a reference pan, and introduced into the calorimeter chamber. The samples were initially cooled from room temperature down to −150 °C. During the subsequent heating scan from −150 °C to 250 °C at a rate of 20 °C/min, the glass transition temperature (Tg) of the non-cross-linked rubber mixture was recorded. At higher temperatures (approximately 130–210 °C), the samples underwent the sulfur vulcanization process directly in the DSC oven, which was manifested as an exothermic peak on the DSC curve. From this recorded exothermic peak, the onset and end temperatures of vulcanization, as well as the enthalpy change (ΔH) of the process, were determined. Nitrogen was used as the protective gas throughout the experiment to prevent oxidation.

### 2.5. Crosslinking Density of the Vulcanizates

The crosslinking density of vulcanizate samples was determined in accordance with the PN-ISO 1817:2021 standard. From each vulcanizate, four specimens were cut to achieve a mass range of 20–50 milligrams. The samples were placed in a weighing vessel, submerged in the solvent (toluen) and left to swell for 48 h. After this period, the samples were weighed, then dried in an oven at 50 °C for 48 h and then reweighed. With this study, the following values can be determined:

Equilibrium Swelling (Equation (2)):

(2) $$Q_{w}=\frac{m_{sp}-m_{s}}{m_{s}}$$

where $Q_{w}$—equilibrium swelling; $m_{sp}$—mass of the swollen sample; $m_{s}$—mass of the dried sample.

- Concentration of the effective chains (Equations (3) and (4)):

(3) $$\gamma_{e}=\frac{{ln(1-V}_{r})+V_{r}+{\mu V}_{r}^{2}}{V_{0}(V_{r}^{\frac{1}{3}}-\frac{V_{r}}{2})}$$

(4) $$V_{r}=\frac{1}{1+Q_{w}\frac{\rho_{k}}{\rho_{r}}}$$

where $\gamma_{e}$—effective crosslinking density [mol/cm^3^]; $V_{r}$—volume fraction of rubber in swollen gel; $V_{0}$—molar volume of the solvent [mol/cm^3^]; $\rho_{k}$—density of the rubber [g/cm^3^]; $\rho_{r}$—density of the solvent [g/cm^3^]; μ—Huggins parameter, equal at 25 °C (Equation (5)):

(5) $$\mu =0.487+(0.228\times V_{r})$$

### 2.6. Mechanical Properties of Vulcanizates Under Static Conditions

The test was conducted using a ZWICK testing apparatus. Three dumbbell-shaped specimens (type W-3, according to the relevant standard), each measuring 4 mm in width, were cut from each vulcanized material. The thickness of each specimen was measured individually (approx. 1 mm) prior to testing and entered into the ZWICK apparatus software (testXpert III, ZwickRoell GmbH & Co. KG, Ulm, Germany). The test parameters were as follows: initial force of 0.3 N and a tensile speed of 500 mm/min.

The study established the following parameters:

- SE100, SE200, SE300—stress at 100%, 200% and 300% elongation [MPa];
- TS—tensile strength [MPa];
- EB—elongation at break [%].

### 2.7. Determination of Hardness of the Vulcanizates

A test was conducted using a ZWICK Shore A hardness tester. The specimens were shaped as discs with dimensions conforming to the standard: 29 mm in diameter and 6 mm in thickness. Five measurements were taken for each specimen by applying a needle with a rounded tip against the test piece. The final result represents the mean value of the obtained measurements.

### 2.8. Thermo-Oxidative and UV-Aging

The vulcanized samples were subjected to a series of treatments, beginning with the meticulous cutting of squares of similar dimensions from each sample. These samples were then placed in a thermo-oxidation chamber set at a temperature of 70 °C. The aging process was conducted over a period of 14 days for each sample.

The UV aging process was carried out using a UV 2000 apparatus from Atlas (Mountainside, NJ, USA), equipped with UV-A lamps emitting at a wavelength of approximately 340 nm. The UV irradiation intensity was maintained at 0.7 W/m^2^, with the lamps positioned about 7 cm from the sample surfaces to ensure uniform exposure. Relative humidity (RH) inside the chamber was maintained at standard laboratory levels (~50–65%). The UV aging was conducted in repetitive cycles simulating natural environmental conditions: a daytime period with UV exposure (intensity = 0.7 W/m^2^, temperature = 60 °C, duration = 8 h) followed by a nighttime period without UV (temperature = 50 °C, duration = 4 h) but with increased humidity to mimic dew formation. The total exposure time in the chamber was 120 h.

The samples were then re-subjected to functional characterization tests in order to assess the degree of degradation that had been caused by the thermal-oxidative and UV-aging process. The calculation of the aging factor (K) was based on the results of the mechanical strength test, which were in turn based on the following Equation (6) [44]:

(6) $$K=\frac{{TS}_{after}\times {EB}_{after}}{{TS}_{before}\times {EB}_{before}}\;$$

where TS_after_—tensile strength after degradation [MPa]; EB_after_—elongation at break after degradation [%]; TS_before_—tensile strength before degradation [MPa]; EB_before_—elongation at break before degradation [%].

### 2.9. Surface Analysis of Vulcanizates Before and After Aging

To evaluate the surface condition of the vulcanizates before and after the aging process, microscopic analysis was performed on the samples. The surface morphology was examined using a Leica MZ6 optical microscope (Leica Microsystems GmbH, Wetzlar, Germany). Images of the sample surfaces were captured at a magnification of 1000×, allowing for detailed observation of structural features and potential surface defects such as microcracks, fissures, or topographical changes.

## 3. Results and Discussion

### 3.1. Rheometric Properties of Elastomeric Blends

**Figure 1:**
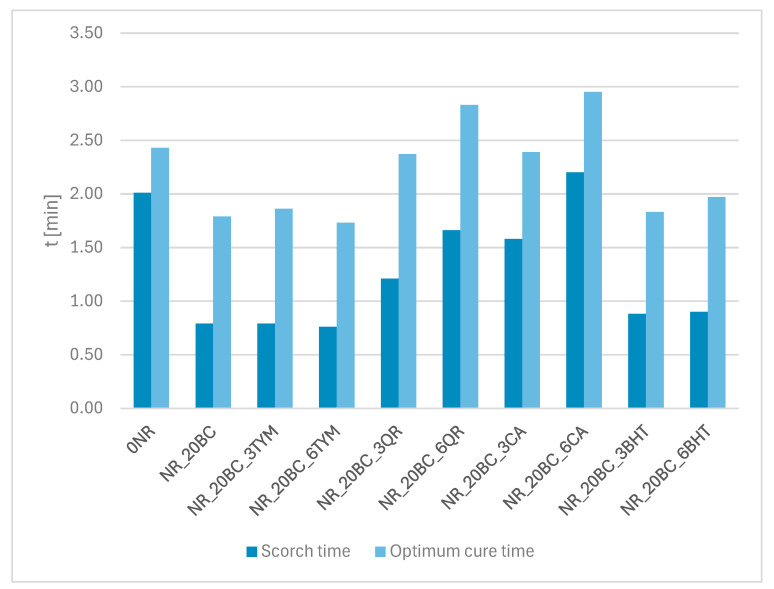
The scorch and optimum cure time of the elastomer mix.

Rheometric analysis was conducted to evaluate the influence of various auxiliary substances, including biochar and selected antioxidants, on the rheological properties of elastomeric mixtures and the course of the vulcanization process. The values of the recorded torque parameters are summarized in Table 2, while the characteristic times describing the vulcanization process are presented in Figure 1.

The incorporation of biochar into natural rubber (sample NR_20BC) resulted in an increase in compound viscosity, as indicated by the higher value of the minimum torque (M_min_) [45], which increased from 0.25 dNm (reference sample NR) to 0.33 dNm. Simultaneously, a significant increase in the maximum torque (M_max_) was observed—from 2.75 to 4.64 dNm—which suggests enhanced stiffness of the vulcanized material and a higher crosslinking density [46]. Additionally, both the scorch time (t_s2_) and the optimum cure time (t_90_) were shortened. This may indicate that biochar, possibly due to the presence of mineral components such as zinc oxide, magnesium oxide, and calcium oxide, plays a critical role in influencing the sulfur vulcanization process of natural rubber [47]. The observed effect of biochar in sulfur vulcanization is primarily attributed to its surface chemistry and physicochemical interactions, which facilitate the formation of sulfur cross-links [20]. Specifically, the high surface area and porous structure of biochar create favorable microenvironments that enhance the adsorption [48] and activation of sulfur species, thereby improving the efficiency and kinetics of cross-linking reactions. In this way, biochar guides and accelerates the vulcanization process by acting as a key influencing factor.

The introduction of antioxidant additives caused varied effects on the rheological behavior of the mixtures. In the case of samples containing thymol (NR_20BC_3TYM and NR_20BC_6TYM), a significant reduction in M_min_ (down to 0.16–0.20 dNm) was recorded, suggesting a plasticizing effect. At the same time, M_max_ values reached the highest among all tested samples (4.84 and 4.83 dNm), which may indicate improved filler dispersion and a favorable influence of thymol on the elastomeric network structure. The scorch and optimum cure times remained similar to those of the biochar-only sample, implying that thymol does not negatively affect the vulcanization kinetics.

Similar behavior was noted for BHT (butylated hydroxytoluene). Samples NR_20BC_3BHT and NR_20BC_6BHT also exhibited low M_min_ values (0.16–0.28 dNm), elevated M_max_ values (4.09–4.23 dNm) and short t_s2_ and t_90_ times. This suggests a positive effect of BHT on maintaining a balance between pre-vulcanization plasticity and post-vulcanization crosslinking.

Contrasting effects were observed for additives such as quercetin (NR_20BC_3QR and NR_20BC_6QR) and caffeic acid (NR_20BC_3CA and NR_20BC_6CA). These samples exhibited a notable decrease in M_max_—approximately 3.5–3.7 dNm for quercetin and 3.0–3.1 dNm for caffeic acid. The reduction in torque and ΔM values indicates a lower degree of crosslinking, which may be attributed to unfavorable interactions of these compounds with the vulcanization system, potentially due to their reducing, antioxidant or chelating properties. By absorbing free radicals, antioxidants prevent excessive crosslinking, resulting in reduced torque requirements during vulcanization. This is further supported by the prolonged scorch and cure times (t_s2_ and t_90_), which, in some cases, exceeded 2.8 min.

Based on the obtained results, it can also be concluded that changing the concentration of additives from 3% to 6% did not cause significant differences in the rheological parameters. For thymol, BHT, quercetin and caffeic acid alike, samples with both lower and higher concentrations behaved very similarly, suggesting that the effect of these substances is achieved already at the lower dose and further increasing the amount does not significantly influence the vulcanization process or the properties of the composites.

### 3.2. Differential Scanning Calorimetry (DSC) Analysis of Elastomeric Composites

The next stage of the study involved determining the influence of anti-aging agents on the temperature and thermal effect of vulcanization of the tested elastomeric mixtures, which were investigated in their uncrosslinked state using differential scanning calorimetry (DSC). Analysis of the results, presented in Table 3, showed that the glass transition temperature did not undergo significant changes and remained within a narrow range from approximately −62.5 °C to −64.4 °C. This indicates that the applied additives do not affect the mobility of polymer chains and, consequently, do not alter the amorphous structure of the elastomer.

Crosslinking (vulcanization) is a key process in the preparation of elastomers, involving the formation of permanent cross-links between polymer chains. This process transforms the linear or branched polymer structure into a three-dimensional network, significantly improving the mechanical, thermal, and chemical resistance properties of the material. Vulcanization affects hardness, tensile strength, elasticity, and aging resistance, making it one of the most important stages in rubber and elastomer processing. Factors such as temperature, curing time, and compound composition have a crucial impact on the course and efficiency of this process. From a scientific perspective, understanding the kinetics of this process is essential, as it enables better insight into reaction mechanisms and optimization of technological parameters.

The addition of biochar to the rubber mixture caused an earlier onset of the vulcanization process—the onset temperature (T_onset_) decreased from 154.8 °C to 148.2 °C and the overall process proceeded with a higher thermal effect (enthalpy increased by approximately 8 J/g compared to the reference sample). The endset temperature (T_endset_) was similar to that of the base sample, suggesting that biochar acts as an activator of the process, facilitating its initiation and intensifying the reaction. These findings align with rheometric data showing increased compound viscosity and faster cure times. In the case of thymol addition, a significant decrease in the onset temperature of vulcanization by about 30 °C was observed, along with an increase in enthalpy by approximately 5 J/g compared to the reference sample. However, at a higher dosage of thymol (6 parts by weight), the enthalpy significantly decreased, which may indicate a nonlinear effect, possibly related to inhibition or alteration of the vulcanization mechanism. Rheometer measurements correspondingly indicated a plasticizing effect at lower doses and altered cure behavior at higher doses. The addition of BHT led to the highest enthalpy among all tested samples, confirming enhanced energy effects during vulcanization. Rheological results support this, showing a favorable balance between compound plasticity and crosslinking density. Conversely, caffeic acid and quercetin exhibited temperatures and enthalpies of the vulcanization process similar to those of the reference sample, indicating their presence does not significantly affect the kinetics. This is consistent with rheometric observations of reduced crosslinking density and prolonged cure times, suggesting mild inhibition of vulcanization.

### 3.3. CrossLinking Density of the Composites

**Figure 2:**
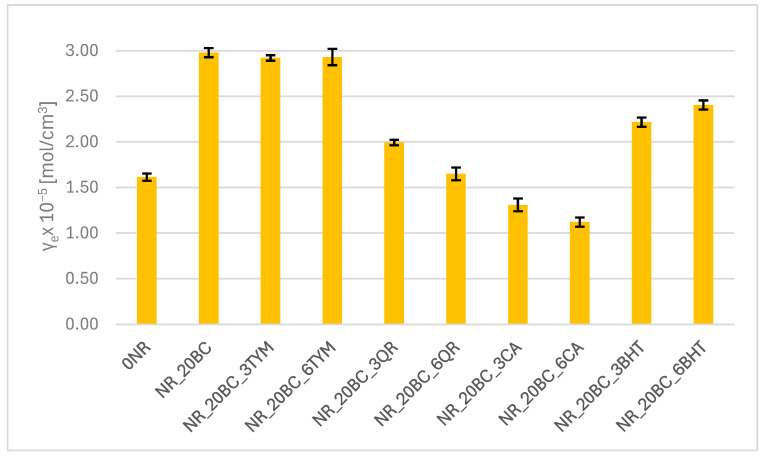
Crosslinking density of vulcanizates.

The reference sample (NR) exhibited a crosslinking density of 1.61 × 10^−5^ mol/cm^3^ (Figure 2). The addition of biochar significantly increased this value to 2.98 × 10^−5^ mol/cm^3^, which is consistent with the previously obtained DSC results, indicating a marked increase in vulcanization enthalpy and a decrease in the onset temperature of the process. These findings are also supported by earlier rheological analyses, which revealed increased stiffness of the compound and shortened vulcanization times. This confirms the activating effect of biochar on the formation of sulfur crosslink networks. Samples containing thymol also demonstrated high crosslinking densities (approximately 2.9 × 10^−5^ mol/cm^3^). The reduction in vulcanization onset temperature at the lower thymol dosage suggests that thymol may promote or facilitate the initiation of crosslinking. At a higher dosage, the process proceeded with lower thermal efficiency; however, the crosslinking density remained high, which may be attributed to the beneficial influence of thymol on filler dispersion and the elastomer network structure. In contrast, samples with quercetin and caffeic acid exhibited a notable reduction in crosslinking density—ranging from 1.99 to 1.65 × 10^−5^ mol/cm^3^ (quercetin) and from 1.31 to 1.12 × 10^−5^ mol/cm^3^ (caffeic acid). These results correlate with the lower enthalpy values obtained from DSC analysis and the prolonged vulcanization times observed in rheometric studies. The presence of these compounds may limit the efficiency of crosslink formation, likely due to their antioxidant and chelating properties. By scavenging free radicals or binding active components of the vulcanization system (e.g., metal ions), they may interfere with the progress of the crosslinking reaction. Samples containing BHT showed a moderate increase in crosslinking density (2.22–2.40 × 10^−5^ mol/cm^3^). The effect of BHT is therefore favorable in terms of elastomer network development, which is in agreement with the observed balance between pre-vulcanization plasticity and post-vulcanization crosslinking, as reflected in rheological measurements.

### 3.4. Mechanical Properties of the Vulcanizates

**Figure 3:**
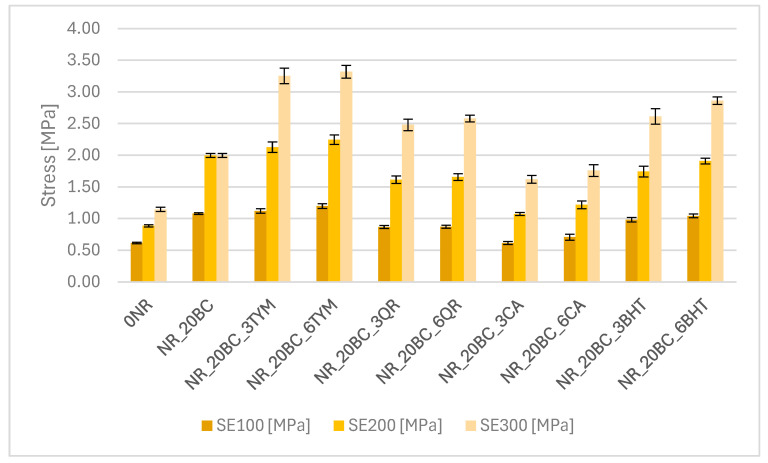
Stress at 100%, 200% and 300% of elongation.

**Figure 4:**
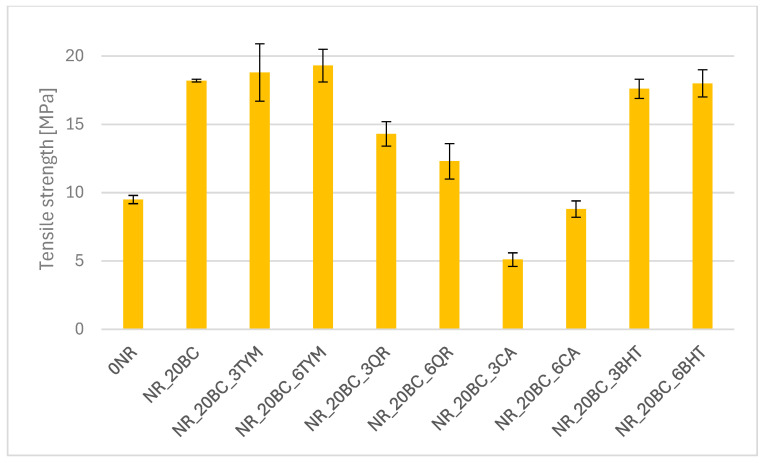
Tensile strength of vulcanizates.

**Figure 5:**
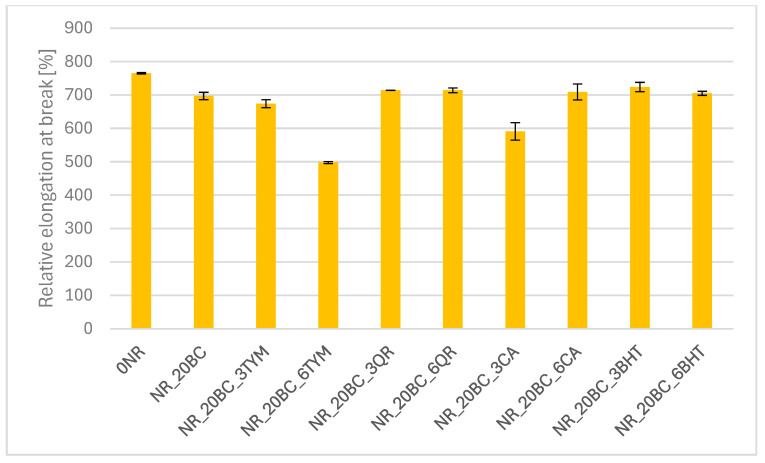
Relative elongation at break of vulcanizates.

The results of tensile strength tests are presented in the form of graphs illustrating the modulus values at 100%, 200% and 300% elongation (Figure 3), tensile strength TS (Figure 4) and elongation at break E_b_ (Figure 5).

The reference sample exhibited the lowest stiffness, (SE_100_—0.61 MPa and SE_300_—1.15 MPa), along with the lowest stress values throughout the deformation range. This is consistent with its low crosslinking density and relatively low torque observed in rheometric tests. The incorporation of biochar resulted in a significant increase in stiffness and strength—SE_100_ increased to 1.08 MPa and TS reached 18.2 MPa. At the same time, a slight decrease in elongation at break (692%) was observed compared to the reference sample (765%), indicating an increase in the rigidity of the elastomer network. These findings correlate with the increased crosslinking density and enhanced vulcanization observed previously. The addition of thymol (NR_20BC_3TYM and NR_20BC_6TYM) led to a further improvement in mechanical performance. The highest stress values at 300% elongation were recorded for the sample with six parts by weight of thymol (3.32 MPa), along with the highest tensile strength (19.3 MPa). However, this was accompanied by a clear reduction in elongation at break (498%), suggesting that higher thymol content promotes stiffness and strength but limits deformability. The favorable dispersion and structuring of the elastomeric network likely contribute to these results. In contrast, the incorporation of quercetin and caffeic acid negatively affected the mechanical properties of the vulcanizates. In both cases, a marked reduction in modulus values and tensile strength was observed. The most pronounced deterioration occurred in the sample with caffeic acid (NR_20BC_3CA), where TS decreased to 5.1 MPa and SE_300_ was only 1.62 MPa. Although quercetin had a slightly less severe effect, the reduction in strength and stiffness was still noticeable. These results are consistent with the lower crosslinking densities and decreased vulcanization enthalpies, likely due to the antioxidant and chelating activity of these compounds, which may inhibit effective crosslink formation. Samples with BHT showed favorable mechanical properties, with high tensile strength (above 17 MPa) and relatively high stiffness, especially at higher elongation levels. Elongation at break remained high (over 700%), suggesting that BHT supports a good balance between elasticity and strength. This is consistent with its moderate impact on the crosslinking density and the favorable course of the vulcanization process observed previously.

### 3.5. Hardness of the Vulcanizates

**Figure 6:**
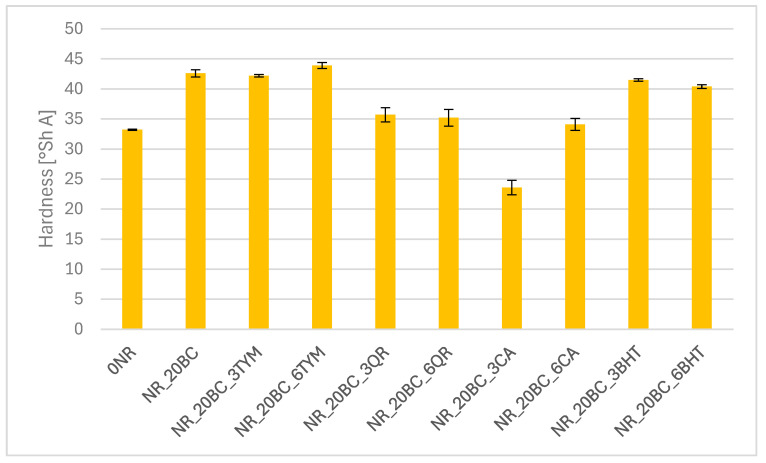
Hardness of vulcanizates.

The hardness of the obtained biocomposites is primarily determined by the crosslinking density of the elastomer matrix and the dispersion of the solid phase. Both factors influence the rigidity and elastic resistance of the material, which is reflected in Shore A hardness values. The results of the hardness measurements are presented in Figure 6.

As anticipated, the reference sample exhibited the lowest hardness value (33.2 ± 0.1), corresponding to its low crosslinking density and absence of fillers. The incorporation of biochar significantly increased hardness to approximately 42.6, confirming its reinforcing character and compatibility with the vulcanization system. Samples additionally modified with thymol or butylated hydroxytoluene retained similarly high hardness values (above 41), indicating favorable effects on network development without compromising surface rigidity. In contrast, the addition of quercetin led to a moderate reduction in hardness, with values around 35, which may be attributed to its partial inhibition of the crosslinking process. The most substantial decrease was observed in systems containing caffeic acid, particularly at the lower concentration, where hardness dropped to 23.6. This result points to a negative impact of caffeic acid on crosslink formation. These findings are consistent with swelling data and thermal analysis, reinforcing the conclusion that both the chemical nature of the additives and the microstructural distribution of fillers critically affect the stiffness and mechanical surface properties of the biocomposites.

### 3.6. Effect of Aging on Crosslinking Density and Mechanical Properties of Composites

The biocomposites were subjected to simulated thermo-oxidative and UV-aging processes to assess their durability and resistance to environmental degradation. During such aging, various phenomena may occur that significantly affect the material’s properties. On the one hand, oxidative degradation can lead to the scission of polymer chains; on the other, secondary crosslinking (post-curing) may take place, i.e., continuation of the vulcanization process through additional chemical reactions triggered by heat, oxygen or UV radiation. These two opposing mechanisms—degradation and post-curing—compete, resulting in structural changes within the polymer network and potentially altering the material’s physicochemical properties. This may manifest as an increase or decrease in crosslinking density, changes in solid phase morphology and modifications of interfacial interactions, all of which influence key mechanical properties such as hardness, elastic modulus, tensile strength and elongation at break. The final effect of aging depends strongly on the type of additives incorporated, particularly their antioxidant properties, radical-scavenging ability and their influence on the progression of secondary crosslinking reactions. To evaluate these changes, crosslinking density, mechanical properties and hardness were remeasured after aging. Additionally, microscopic analysis of the sample surface morphology was performed.

**Figure 7:**
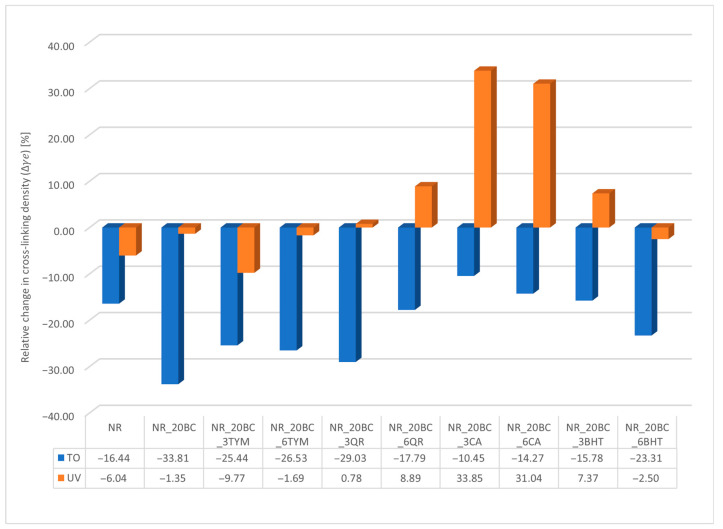
Relative change in crosslinking density after thermo-oxidative and ultraviolet aging.

The changes in crosslinking density of the composites due to thermo-oxidative aging and UV radiation, resulting from various processes occurring within the material structure, are presented in Table 4, while the relative changes after the aging processes are illustrated in Figure 7.

The greatest decrease in crosslinking density after thermo-oxidative aging was observed in the composite containing biochar (NR_20BC), where the νe value decreased by as much as 33.81%. This significant reduction results from the presence of transition metal elements in the biochar, which promote oxidation reactions by accelerating the generation of reactive oxygen species and the cleavage of sulfur crosslinks. Consequently, the three-dimensional network structure degrades and the material loses chemical stability, especially under elevated temperature and oxygen exposure. The use of phenolic antioxidants such as thymol and BHT markedly mitigated the harmful effects of thermo-oxidative aging—in samples containing these additives, the decrease in crosslinking density was approximately 15%. Their mechanism of action is based on scavenging oxygen-centered free radicals (RO^•^, ROO^•^), thereby interrupting the chain oxidation reactions and protecting the network structure. Moreover, after UV-aging, an increase in νe was observed, indicating controlled secondary crosslinking without significant structural deterioration. Natural phenolic compounds, such as caffeic acid and quercetin, exhibited a dual protective function: they neutralized free radicals and chelated metal ions that catalyze oxidation reactions. This enabled them to limit polymer chain degradation. After UV exposure, samples containing these compounds showed a substantial increase in crosslinking density (e.g., +33.85% for NR_20BC_3CA), suggesting their involvement in photochemical crosslinking processes or radical stabilization leading to the formation of new crosslinking bridges. In summary, the durability of the elastomeric network structure under aging conditions strongly depends on the protective agents used. Properly selected antioxidants and UV stabilizers effectively limit oxidative degradation, preserve sulfur crosslinks and may promote secondary crosslinking, thereby enhancing the long-term stability of the material. In contrast, the absence of effective stabilization, as in the case of the biochar-only composite, results in significant degradation and loss of mechanical properties. These observations are corroborated by the results presented in Table 5.

**Figure 8:**
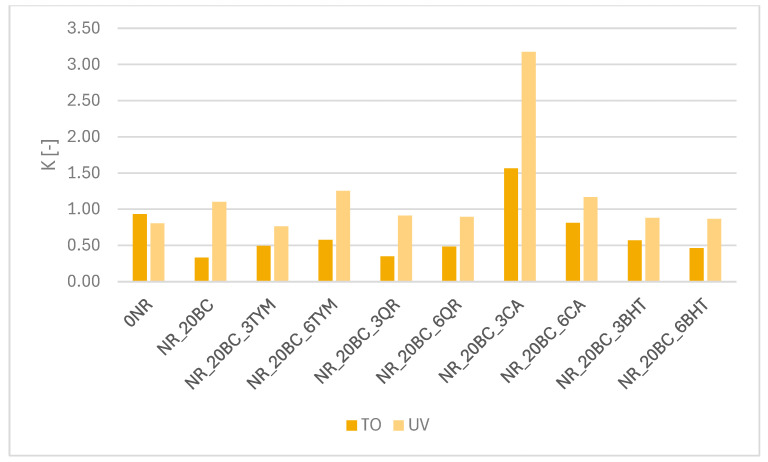
Aging coefficient (K) of rubber composites after thermo-oxidative (TO) and ultraviolet (UV) aging.

The results of mechanical properties presented in the table, combined with the analysis of the K coefficient (Figure 8), clearly confirm the impact of aging processes and the effectiveness of applied anti-aging additives on the stability of elastomer composites. The K coefficient, calculated as the ratio of tensile strength (TS) after aging to its value before aging, serves as a numerical measure of mechanical property changes—the closer the K value is to 1, the smaller the changes, indicating greater material resistance to degradation. Values significantly below 1 reflect strength loss, while values above 1 may suggest secondary crosslinking phenomena.

The reference sample (0NR), containing neither filler nor protective additives, showed a slight decrease in tensile strength after thermo-oxidative aging (from 9.5 to 9.1 MPa) and a moderate change in the K value, indicating a relatively stable NR matrix structure under oxidative conditions. After UV-aging, TS decreased to 7.5 MPa, with a slight increase in elongation at break, possibly due to secondary crosslinking processes taking place within the matrix. A contrasting situation was observed in the sample containing only biochar (NR_20BC). Following thermo-oxidative aging, TS dropped drastically from 18.2 to 7.8 MPa, corresponding to an exceptionally low K value (~0.43). This drop reflects severe degradation of the network structure, in line with the previously observed reduction in crosslinking density (−33.81%). Conversely, after UV exposure, TS increased to 19.6 MPa and the K value exceeded 1, suggesting that secondary crosslinking occurred—although likely unevenly, potentially resulting in localized stiffening of the material. The addition of antioxidants such as thymol significantly limited degradation and improved the composites’ aging resistance. In NR_20BC_3TYM and NR_20BC_6TYM, TS values after thermo-oxidative aging remained at 10.9 and 9.9 MPa, respectively, with K values around 0.6–0.7, indicating reduced mechanical loss. Upon UV exposure, TS increased to 14.5 and 17.7 MPa and K values exceeded 0.9, reflecting thymol’s beneficial effect on secondary crosslinking and structural stabilization. Similar, though slightly weaker, protective effects were observed for quercetin. NR_20BC_3QR and NR_20BC_6QR samples, despite reductions in TS after thermo-oxidative aging (to 6.5–7.1 MPa), retained relatively high elongation and showed K values of about 0.5–0.6 (TOA) and ~0.9 (UV). After UV exposure, TS increased to 13.4 and 11.8 MPa, confirming quercetin’s effectiveness in limiting degradation and promoting photochemical crosslinking. The most distinctive behavior was observed for samples containing caffeic acid, especially NR_20BC_3CA, which despite its low initial TS (5.1 MPa), showed an increase to 7.7 MPa after thermo-oxidative aging (K > 1) and to 13.5 MPa after UV exposure (K > 3). This significant increase confirms strong, controlled UV-induced secondary crosslinking. Similarly, NR_20BC_6CA demonstrated excellent mechanical stability, with K values exceeding 1.5 (UV) and around 1 (TOA). The synthetic antioxidant BHT provided the highest structural stability. NR_20BC_3BHT and NR_20BC_6BHT samples maintained TS values of 11.8 and 9.7 MPa, respectively, after thermo-oxidative aging, with K values around 0.6–0.7. Following UV-aging, TS increased to 15.8–16.0 MPa and K values approached 0.9–1, confirming BHT’s ability to protect the elastomer network against both oxidative and UV degradation while also supporting effective secondary crosslinking without significant loss of flexibility. In addition to mechanical strength and elongation, hardness was also evaluated as an indicator of changes in the material structure during aging (Table 6). In most cases, a slight increase in hardness was observed, particularly after UV exposure, which further supports the hypothesis of additional crosslink formation. This trend is consistent with the observed increases in crosslinking density and K values, especially in systems containing active stabilizers, indicating progressive network densification under aging conditions.

In summary, the mechanical performance results, supported by K coefficient and hardness analysis, clearly demonstrate that the effectiveness of anti-aging additives strongly determines the resistance of elastomer composites to degradation. The composite containing only biochar showed the highest susceptibility to structural deterioration, while additives such as BHT, thymol, caffeic acid and quercetin significantly limited oxidative degradation, promoted secondary crosslinking and helped retain or even improve the mechanical properties of the materials under environmental stress conditions.

### 3.7. Effect of Aging on the Surface Structure of Composites

**Figure 9:**
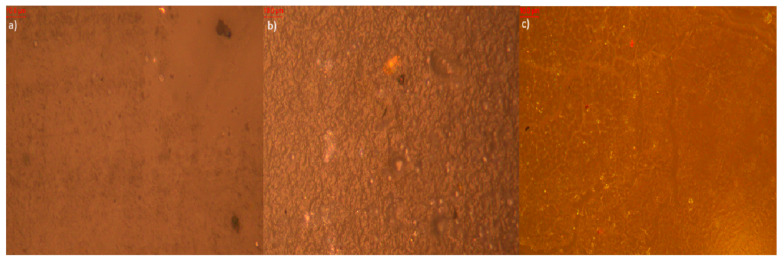
Photos of the reference sample (**a**) before aging (approx. 1000× magnification); (**b**) after thermo-oxidative aging (approx. 1000× magnification); (**c**) after UV-aging (approx. 1000× magnification).

**Figure 10:**
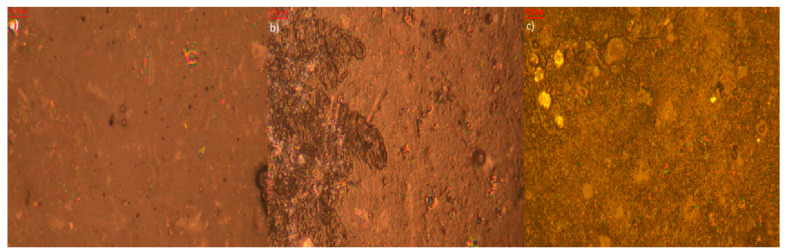
Photos of the sample filled with biochar (**a**) before aging (approx. 1000× magnification); (**b**) after thermo-oxidative aging (approx. 1000× magnification); (**c**) after UV-aging (approx. 1000× magnification).

**Figure 11:**
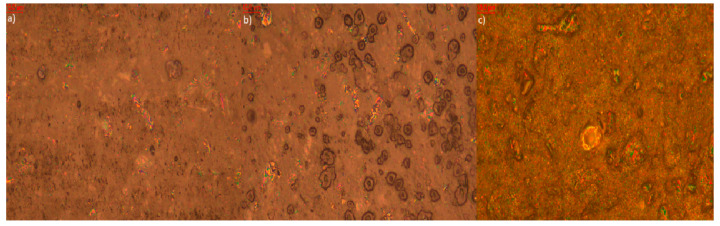
Photo of a sample with the addition of 6 parts by weight of thymol (**a**) before aging (approx. 1000× magnification); (**b**) after thermo-oxidative aging (approx. 1000× magnification); (**c**) after UV-aging (approx. 1000× magnification).

**Figure 12:**
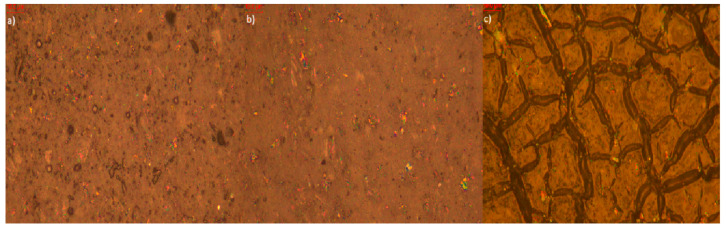
Photo of a sample with the addition of 6 parts by weight of quercetin (**a**) before aging (approx. 1000× magnification); (**b**) after thermo-oxidative aging (approx. 1000× magnification); (**c**) after UV-aging (approx. 1000× magnification).

**Figure 13:**
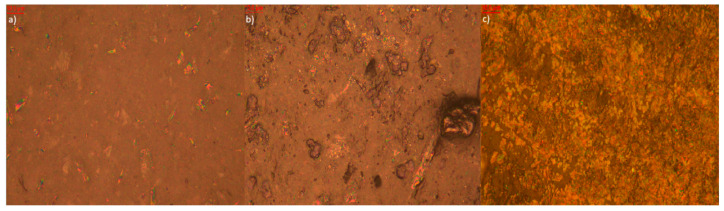
Photo of a sample with the addition of 6 parts by weight of caffeic acid (**a**) before aging (approx. 1000× magnification); (**b**) after thermo-oxidative aging (approx. 1000× magnification); (**c**) after UV-aging (approx. 1000× magnification).

**Figure 14:**
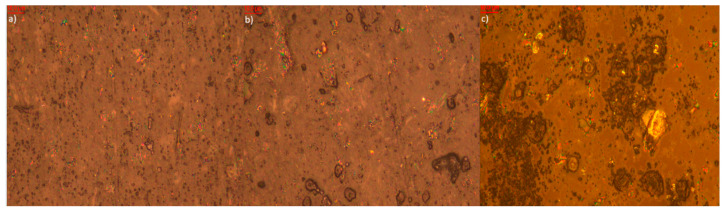
Photo of a sample with the addition of 6 parts by weight of BHT (**a**) before aging (approx. 1000× magnification); (**b**) after thermo-oxidative aging (approx. 1000× magnification); (**c**) after UV-aging (approx. 1000× magnification).

Microscopic analysis of elastomer samples before and after artificial aging (Figure 9, Figure 10, Figure 11, Figure 12, Figure 13 and Figure 14) reveals distinct differences in surface morphology, reflecting the effectiveness of the applied stabilizers in limiting material degradation.

The reference sample without additives (Figure 9) initially exhibits a smooth and homogeneous surface. After thermo-oxidative aging, evident cracking and increased roughness appear, indicating oxidative degradation. Exposure to UV radiation leads to further surface damage—discoloration and irregularities—confirming the high susceptibility of the elastomeric matrix to degradation in the absence of protective agents.

The sample containing biochar (Figure 10) displays increased roughness even prior to aging due to the presence of the filler. After thermo-oxidative aging, the surface becomes deeply cracked with clear signs of degradation. UV-aging results in further discoloration and fragmentation of the surface. Additionally, surface efflorescence is observed, originating from both the biochar itself and its degradation products. This may indicate migration and reactivity of mineral components inherent in the filler structure.

A markedly different effect is seen in the sample with 6 parts by weight of thymol (Figure 11), which maintains a compact and uniform morphology throughout the aging processes. Only minor changes are observed after thermo-oxidative aging and after UV exposure, the surface remains practically intact—with no significant cracking or discoloration. However, small deposits of the aging inhibitor are visible on the surface, suggesting partial migration of thymol. Nevertheless, its presence significantly improves the resistance of the composite to degradation processes.

The sample with six parts by weight of quercetin (Figure 12) initially shows a smooth surface. After thermo-oxidative aging, moderate surface roughness develops, but the most intense changes occur following UV exposure—prominent cracking and discoloration are visible. Surface efflorescence is also present, likely due to partial migration of quercetin to the surface of the composite. This suggests that while quercetin may reduce oxidation, its effectiveness under UV radiation is lower compared to thymol.

A similar trend is observed in the sample with caffeic acid (Figure 13). Before aging, the surface appears uniform; after thermal aging, moderate damage is noted, while UV- aging results in pronounced cracking and discoloration. Surface deposits can also be seen, originating either from caffeic acid or its degradation by-products. Despite its known antioxidant properties, caffeic acid appears less effective in UV conditions than BHT or thymol.

In contrast, the sample with BHT (Figure 14) maintains a stable and uniform surface structure regardless of the aging type. Post-aging changes are minimal—no significant cracking or discoloration is observed and surface efflorescence is negligible, indicating better retention and stability of the additive in the elastomer matrix. This confirms BHT’s high efficacy as a stabilizer against both oxidative and photochemical degradation.

In summary, microscopic observations align with mechanical and chemical test results, clearly indicating that the type and presence of stabilizers significantly influence the durability of elastomer composites. Biochar alone accelerates degradation and shows visible surface deposits, while the presence of antioxidants particularly thymol and BHT effectively protects the surface structure, delays damage development due to aging and minimizes migration of components to the material’s surface.

## 4. Conclusions

Based on the analyses of physicochemical, mechanical properties and microscopic observations, it can be unequivocally concluded that the stability of elastomer composites subjected to artificial aging processes (thermo-oxidative and UV exposure) is strongly dependent on the applied anti-aging additives. Although biochar serves as a reinforcing filler, it also promotes material degradation—both by facilitating oxidative reactions (due to the presence of transition metal oxides) and by lacking radical-scavenging capacity. As a result, significant decreases in crosslinking density (νe), tensile strength (TS) and elongation at break (E_b_) were observed, along with visible surface defects and blooming.

The incorporation of antioxidant additives significantly improved the resistance of the materials to degradation. Both the synthetic stabilizer BHT and natural phenolic compounds such as thymol, caffeic acid and quercetin helped mitigate the loss of mechanical properties and stabilized the elastomer network structure. Particularly effective were BHT and thymol, as indicated by their high K coefficient values—a numerical measure of the preservation of mechanical performance as well as the preserved surface integrity of aged samples. Moreover, the presence of natural antioxidants enabled photo-induced secondary crosslinking (especially under UV exposure), which, in some cases, resulted in increased crosslinking density and partial recovery of mechanical properties. It was also noted that some additives (e.g., quercetin and caffeic acid) may partially migrate to the surface, leading to blooming, which could limit their long-term stabilizing efficacy.

In summary, the appropriate selection of anti-aging additives is critical for ensuring the durability, structural integrity and long-term functionality of elastomer composites under environmental aging conditions. Stabilizers capable of effectively scavenging free radicals and suppressing oxidation reactions not only protect the material but may also promote beneficial secondary crosslinking processes, ultimately enhancing the longevity and performance of rubber-based products.

## Figures and Tables

**Table 1:** Composition of rubber mixtures.

Ingredients	Quantity [phr]		
	Reference Mixture	Benchmark Mixture	Tested Mixture
NR	100	100	100
ZnO	5	5	5
Sulfur	2	2	2
MBT	2	2	2
Stearin	1	1	1
Biochar	-	20	20
Anti-Aging Substances	-	-	3/6

**Table 2:** Rheometric properties of elastomer mixtures.

Sample	M_min_ [dNm]	M_max_ [dNm]	ΔM [dNm]
0NR	0.25	2.75	2.50
NR_20BC	0.33	4.64	4.31
NR_20BC_3TYM	0.16	4.84	4.68
NR_20BC_6TYM	0.20	4.83	4.63
NR_20BC_3QR	0.20	3.72	3.52
NR_20BC_6QR	0.28	3.51	3.23
NR_20BC_3CA	0.17	3.14	2.97
NR_20BC_6CA	0.32	3.04	2.72
NR_20BC_3BHT	0.28	4.09	3.81
NR_20BC_6BHT	0.16	4.23	4.07

**Table 3:** The thermal characteristics of elastomeric composites determined by DSC analysis, including key parameters such as initial (T_onset_) and final (T_endset_) vulcanization temperatures, enthalpy change (ΔH) and glass transition temperature (T_g_).

Sample	[T_onset_] [°C]	[T_endset_] [°C]	[ΔH] [J/g]	[T_g_] [°C]
0NR	154.8	210.4	9.8	−62.6
NR_20BC	148.2	216.3	17.1	−62.9
NR_20BC_3TYM	111.9	228.9	22.8	−63.7
NR_20BC_6TYM	156.9	208.4	9.5	−63.1
NR_20BC_3QR	154.6	206.5	18.9	−63.7
NR_20BC_6QR	159.2	203.0	14.5	−63.1
NR_20BC_3CA	162.6	204.5	20.1	−62.5
NR_20BC_6CA	158.9	203.5	21.9	−64.4
NR_20BC_3BHT	121.1	224.6	31.6	−63.3
NR_20BC_6BHT	123.7	209.4	27.1	−62.6

**Table 4:** Crosslinking density of vulcanizates before and after thermo-oxidative (TO) and ultraviolet (UV) aging.

Sample	Before Aging	TO	UV
	$\gamma_{e}$·10^−5^ [mol/cm^3^]		
NR	1.61 ± 0.04	1.35 ± 0.05	1.52 ± 0.02
NR_20BC	2.98 ± 0.05	2.19 ± 0.10	3.06 ± 0.08
NR_20BC_3TYM	2.92 ± 0.03	2.18 ± 0.06	2.57 ± 0.07
NR_20BC_6TYM	2.93 ± 0.09	2.15 ± 0.08	2.88 ± 0.06
NR_20BC_3QR	1.99 ± 0.03	1.41 ± 0.04	2.01 ± 0.05
NR_20BC_6QR	1.65 ± 0.07	1.36 ± 0.08	1.80 ± 0.09
NR_20BC_3CA	1.31 ± 0.07	1.17 ± 0.03	1.75 ± 0.08
NR_20BC_6CA	1.12 ± 0.05	0.96 ± 0.03	1.47 ± 0.02
NR_20BC_3BHT	2.22 ± 0.05	1.87 ± 0.06	2.38 ± 0.07
NR_20BC_6BHT	2.40 ± 0.05	1.80 ± 0.04	2.34 ± 0.07

**Table 5:** The effect of thermo-oxidative (TO) and ultraviolet (UV) aging on the mechanical properties of vulcanizates.

Sample	Before Aging		TO		UV	
	TS [Mpa]	E_b_ [%]	TS [Mpa]	E_b_ [%]	TS [Mpa]	E_b_ [%]
0NR	9.5 ± 0.3	765 ± 2	9.1 ± 0.1	741 ± 9	7.5 ± 0.1	776 ± 3
NR_20BC	18.2 ± 0.1	697 ± 11	7.8 ± 2.1	529 ± 7	19.6 ± 0.3	701 ± 6
NR_20BC_3TYM	18.8 ± 2.1	674 ± 12	10.9 ± 0.3	573 ± 12	14.5 ± 0.5	667 ± 4
NR_20BC_6TYM	19.3 ± 1.2	498 ± 3	9.9 ± 0.8	562 ± 18	17.7 ± 1.0	682 ± 13
NR_20BC_3QR	14.3 ± 0.9	714 ± 1	6.5 ± 0.4	546 ± 9	13.4 ± 1.0	692 ± 17
NR_20BC_6QR	12.3 ± 1.3	714 ± 7	7.1 ± 0.1	592 ± 6	11.8 ± 0.8	666 ± 7
NR_20BC_3CA	5.1 ± 0.5	591 ± 26	7.7 ± 0.3	604 ± 4	13.5 ± 0.7	697 ± 18
NR_20BC_6CA	8.8 ± 0.6	709 ± 24	7.8 ± 0.1	650 ± 10	10.6 ± 0.4	688 ± 2
NR_20BC_3BHT	17.6 ± 0.7	724 ± 14	11.8 ± 0.5	616 ± 23	15.8 ± 0.4	708 ± 17
NR_20BC_6BHT	18 ± 1.0	705 ± 6	9.7 ± 0.1	606 ± 14	16.0 ± 1.6	687 ± 16

**Table 6:** Hardness of the vulcanizates before and after aging.

Sample	Before Aging	TO	UV
	°Sh A		
0NR	33.2 ± 0.1	28.9 ± 0.1	29.0 ± 0.6
NR_20BC	42.6 ± 0.6	40.6 ± 0.2	45.4 ± 0.6
NR_20BC_3TYM	42.2 ± 0.2	40.64 ± 0.6	48.4 ± 0.04
NR_20BC_6TYM	43.9 ± 0.5	43.1 ± 0.6	45.1 ± 1.6
NR_20BC_3QR	35.7 ± 1.2	38.9 ± 0.6	41.7 ± 1.3
NR_20BC_6QR	35.2 ± 1.4	32.9 ± 0.9	43.8 ± 0.5
NR_20BC_3CA	23.6 ± 1.2	31.5 ± 0.6	39.7 ± 0.4
NR_20BC_6C	34.1 ± 1.0	28.7 ± 0.6	38.1 ± 0.3
NR_20BC_3BHT	41.5 ± 0.2	41.2 ± 0.6	41.4 ± 0.2
NR_20BC_6BHT	40.4 ± 0.3	36.1 ± 0.5	43.5 ± 0.3

## Data Availability

The raw data supporting the conclusions of this article will be made available by the authors on request.
